# Minimally invasive computer-navigated total hip arthroplasty, following the concept of femur first and combined anteversion: design of a blinded randomized controlled trial

**DOI:** 10.1186/1471-2474-12-192

**Published:** 2011-08-19

**Authors:** Tobias Renkawitz, Martin Haimerl, Lars Dohmen, Sabine Gneiting, Melanie Wegner, Nicole Ehret, Claudia Buchele, Mario Schubert, Philipp Lechler, Michael Woerner, Ernst Sendtner, Tibor Schuster, Kurt Ulm, Robert Springorum, Joachim Grifka

**Affiliations:** 1 Department of Orthopaedic Surgery, Regensburg University Medical Center, Germany; 2 Brainlab AG, R&D Surgery, Feldkirchen, Germany; 3 Institute for Medical Statistics and Epidemiology, TU Muenchen, Germany

## Abstract

**Background:**

Impingement can be a serious complication after total hip arthroplasty (THA), and is one of the major causes of postoperative pain, dislocation, aseptic loosening, and implant breakage. Minimally invasive THA and computer-navigated surgery were introduced several years ago. We have developed a novel, computer-assisted operation method for THA following the concept of "femur first"/"combined anteversion", which incorporates various aspects of performing a functional optimization of the cup position, and comprehensively addresses range of motion (ROM) as well as cup containment and alignment parameters. Hence, the purpose of this study is to assess whether the artificial joint's ROM can be improved by this computer-assisted operation method. Second, the clinical and radiological outcome will be evaluated.

**Methods/Design:**

A registered patient- and observer-blinded randomized controlled trial will be conducted. Patients between the ages of 50 and 75 admitted for primary unilateral THA will be included. Patients will be randomly allocated to either receive minimally invasive computer-navigated "femur first" THA or the conventional minimally invasive THA procedure. Self-reported functional status and health-related quality of life (questionnaires) will be assessed both preoperatively and postoperatively. Perioperative complications will be registered. Radiographic evaluation will take place up to 6 weeks postoperatively with a computed tomography (CT) scan. Component position will be evaluated by an independent external institute on a 3D reconstruction of the femur/pelvis using image-processing software. Postoperative ROM will be calculated by an algorithm which automatically determines bony and prosthetic impingements.

**Discussion:**

In the past, computer navigation has improved the accuracy of component positioning. So far, there are only few objective data quantifying the risks and benefits of computer navigated THA. Therefore, this study has been designed to compare minimally invasive computer-navigated "femur first" THA with a conventional technique for minimally invasive THA. The results of this trial will be presented as soon as they become available.

**Trial registration number:**

DRKS00000739

## Background

One of the great intraoperative challenges in total hip arthroplasty (THA) is to find an optimized compromise between hip biomechanics, tribology and post-operative functionality. Component malpositioning and soft tissue imbalance influence the prevalence of dislocation, loosening, wear, containment, postoperative range of motion (ROM) and impingement. Impingement can be caused through component-to-component contact (periprosthetic impingement), bone-to-bone contact (bony impingement) or component-to-bone contact (bone-to-prosthesis impingement). Bone-to-bone impingement is usually correlated to the offset of the prosthesis while component-to-component impingement is highly dependent on the design and position of the total joint components. The surgeon's intraoperative task is to weigh stable cup containment against optimal postoperative ROM with no impingement [1-6]. Traditionally, THA begins with the preparation of the acetabulum and implantation of the prosthetic cup, followed by the preparation of the femoral cavity and stem insertion. In cementless THA performed manually, the antetorsion of the femoral stem is usually hard to control. Depending on the anatomical shape of the femur, the broaches and the definitive implant virtually "find their way" to a rotational position, where the stem conforms best to the rigid shape of the native proximal femur canal. This results in a wide variability of stem antetorsion from 15° of retroversion to 45° of anteversion on the postoperative CT scans [1,7,8]. In contrast, cup inclination and cup anteversion can be controlled to a certain extent by the surgeon during the reaming and implantation process. Multifold models have been developed to determine the optimal combination of cup inclination, cup anteversion, and stem antetorsion for maximizing ROM and minimizing the risk for impingement. In this context, different authors have proposed starting with the preparation of the femur ("femur first") and then adjusting the position of the cup in accordance to the femoral rotation. For a secure component fixation and orientation, the sum of the stem antetorsion and cup anteversion, known as "combined (ante)version", must then fall within one of the following criteria: close to 45° for women/between 20°-30° for men, according to Ranawat; between 40°-60°, according to Jolles; between 25°-50°, according to Dorr; or it should satisfy the equation give by Widmer, i.e. cup anteversion + (0.7 × stem antetorsion) = 37.3° [2,9-11]. This complementary component orientation should ensure proper alignment of the femoral head within the cup without impingement of the two throughout all body positions, and has been referred as the "zone of compliance" [11].

In the past, computer navigation has improved the accuracy of component positioning. Non-image based or imageless navigation systems, which do not require additional pre- or intra-operative image acquisition, have demonstrated their ability to significantly reduce the variability in positioning the acetabular component and have shown to precisely measure leg length and offset changes during THA [12-15]. Nowadays, these navigation systems also have the prowess to couple three dimensional simulations with real time evaluations of surgical performance [16].

Hence, the purpose of this study is to conduct a patient- and observer-blinded, randomized controlled trial with a newly developed computer-assisted operation method for THA following the concept of "femur first"/"combined anteversion," which includes various aspects of a functional optimization of the cup position and comprehensively addresses ROM as well as containment and alignment parameters. It is our hypothesis that minimally invasive computer-navigated "femur first" THA will lead to an increased ROM without bony or periprosthetic impingement. The present paper reports on the methodological design of the study.

## Methods/Design

### Study design

A patient- and observer-blinded randomized controlled trial will be conducted. Patients will be randomly allocated to receive minimally invasive computer-navigated "femur first" THA or the conventional minimally invasive THA procedure. The random allocation sequence will be computer-generated in a permuted block randomization design by one of the associate statisticians (TS, KU) using certificated randomization software (Rancode 3.6 Professional, IDV, Gauting, Germany). Permuted blocks of size 4, 6 and 8 participants will be employed to ensure a balanced allocation sequence. This sequence will then be placed into sealed, consecutively numbered, opaque envelopes. These envelopes will be kept in a locked filing cabinet in the office of the surgeon who will open the envelopes in order of participant recruitment. The random allocation sequence will not be revealed to the clinical examiners conducting the baseline or follow-up assessments and will only be opened by the study surgeon upon notification of completion of the baseline assessment by the clinical examiner.

The study design, procedures and informed consent are approved by the Medical Ethics Committee of Regensburg University Medical Center (No.: 10-121-0263). The trial is registered at the German Clinical Trials Register and the registry platform for international clinical trials of the World Health Organization (WHO) under the same Main ID DRKS00000739.

### Study population

The study will be conducted at the Orthopedic Department of Regensburg University Center, Asklepios Klinikum Bad Abbach, Germany. Patients between the ages of 50 and 75 with an American Society of Anesthesiologists (ASA) score ≤ 3 who are admitted for primary cementless unilateral THA due to primary or secondary osteoarthrosis will be included. Participation in the study is voluntary and patients have to provide informed consent (incl. consent to the postoperative computed-tomography scan) before participation. Exclusion criteria include an ASA score > 4, pregnancy, THA on the contralateral side and inability to understand the consent form. The inclusion period is planned from June 2011 to August 2012.

### Intervention

Both groups will have surgery using the minimally invasive single-incision Micro-Hip^® ^approach in a lateral-decubitus position [17]. In brief, the surgical approach begins with an incision midway along the greater trochanter on its ventral edge and runs for 6-8 cm in the direction of the anterior superior iliac spine. Then, the subcutis is severed, revealing the iliotibial tract and the fascia. After incision of the fascia, the approach follows the interval between the tensor fascia lata muscle and the rectus muscle using a section of the anterior iliofemoral approach described by Smith-Petersen. No tendon or muscle is cut or detached. The joint capsule is split and left in place. The hip joint is not dislocated, the osteotomy of the femoral neck is performed in situ. The head is then removed. An angulated, minimally invasive instrument with a double offset will be used in all operations in order to facilitate muscle sparing reaming and insertion of the cup and stem. The leg is extended, adducted and externally rotated in order to prepare the femur. After final reposition the fascia and skin are closed [17]. Press-fit acetabular components and cement-free hydroxyapatite-coated stems (Pinnacle cup, Corail stem, DePuy, Warsaw, IN, USA) with metal heads will be used in both groups. The intra- and postoperative definition of the acetabular planes for cup inclination and anteversion will rely on the same Murray (radiographic) plane and coordinate system in both groups [18].

#### Minimally invasive, computer-navigated femur first (FF) THA

In the minimally invasive computer-navigated "femur first" (FF) THA group, an imageless navigation system (BrainLAB Navigation System Prototype Hip 6.0 "Femur First", Feldkirchen, Germany) with a newly developed "femur first" prototype software will be used. For the navigation process, reference pins (two Kirschner wires, 3.2 mm diameter) will be inserted into the anterior iliac crest and into the ventro-lateral third of the distal femur after stab incisions are made. Dynamic reference bases will be then attached to the pins. As a next step, the anterior superior iliac spine (ASIS) and pubic tubercle points will be registered using a reference pointer positioned on the skin surface. These points define the reference coordinate system of the pelvis, i.e. anterior pelvic plane and midsagittal plane as the symmetry plane of both ASIS points. On the femoral side, the medial and lateral aspect of the epicondyles and ankle points will be registered. The knee has to be flexed by 90° during the acquisition of the ankle points. These points define the center of the condyles as well as ankle. After osteotomy of the femoral neck and removal of the head, the femur will be exposed. Points at the femoral resection plane will be registered for assessment of bony range of motion. Reaming of the femoral medullary canal will incorporate various measurements of the femoral anatomy using the navigation system, including the entry point of the proximal shaft and the antetorsion. Then, the acetabular anatomy will be registered and reamed. Depending on the information gathered during the femoral and acetabular preparation, the navigation system now calculates an optimized, impingement-free cup position which is presented to the surgeon on a screen. After the insertion of the uncemented acetabular cup in this prescribed position, the uncemented femoral component is placed, followed by placement of a head on the femoral component, repositioning of the joint and closure in layers. All landmark and verification information will be logged by the navigation system for further analysis steps.

#### Conventional minimally invasive THA

In the conventional minimally invasive THA group, the preparation of the acetabulum and implantation of the prosthetic cup will be followed by the preparation of the femoral cavity and stem insertion. Acetabular components will be placed free-hand without the aid of mechanical or computerised alignment guides. Our target acetabular component position for all patients lie within the "safe zone" as defined by Lewinnek et al. (40° ± 10° inclination and 15 ± 10°, anteversion, as estimated visually by the surgeon) [19]. Since the patients will be blinded to the allocated intervention, the same stab incision on the anterior iliac crest and the ventro-lateral third of the distal femur are made as for the computer-navigated FF THA group.

### Measurements

#### Clinical evaluation

For a pre- and post-operative clinical examination, a selection of the most widely used questionnaires in THA research will be used [20,21]. The validated Hip Osteoarthritis Outcome Score (HOOS) and the Harris Hip Score will be used as a disease-specific outcome instrument [22,23]. The SF-36 and Euro-Qol 5D are generic questionnaires and will be used to measure health-related quality of life. Patients' satisfaction with the results of the surgical procedure will be measured with the Patient Satisfaction Scale [24]. Perioperative and postoperative complications will be registered. A pre- and post-operative gait analysis using a gait laboratory and a musculoskeletal model will be performed for a subset of patients. Hip reaction forces, hip angle variation, implant-bone contact stress and muscle forces/activation will be evaluated.

#### Radiographic evaluation

Postoperatively, a pelvic/femoral computed tomography (CT) scan will be performed. The position of the acetabular component will be evaluated by an independent external institute (MeVis, Bremen, Germany) on a 3D reconstruction of the pelvis using image-processing software (based on MeVisLab, MeVis). The postoperative range of motion/ROM of the treated hip joint until bony or metal impingement will then be determined. For this purpose, the bone structures will be segmented in the post-operative CT data set. Additionally, reference landmarks for providing the pelvic and femoral coordinate system are defined. This includes both ASIS and pubic tubercle points for the pelvic coordinate system. The segmentation of the bones and definition of the landmarks will be performed by the same external institute (MeVis, Bremen, Germany) which is blinded to individual patient data including the assignment to one of the groups. Based on the segmented bone models, the postoperative ROM will be calculated by an algorithm which automatically determines bony and prosthetic impingements. The accuracy of the image-free navigation procedure for cup and stem placement and the surgeons' intraoperative estimates will be assessed by comparing the intraoperatively acquired implant positions with component measurements on the postoperative CT data sets.

#### Perioperative measurements

Intraoperatively, average surgical time, surgeons' intraoperative estimates for acetabular and femoral component position, and intraoperative blood loss will be documented. Postoperatively, in-hospital transfusion rate, wound healing disorders, length of hospital stay, and daily pain on a visual analogue pain scale (0-10) will be recorded until discharge.

#### Primary endpoint definition and sample size justification

Sample size calculation is based on the primary hypothesis that minimally-invasive computer-navigated FF THA group will lead to an increased ROM with no impingement. Accordingly, the primary endpoint to be comparatively assessed in this study will be the proportion of patients showing post-surgery optimal ROM configurations without osseous or metallic impingement. The Chi-square test will be used for statistical assessment of the primary study hypothesis. In order to detect differences of at least 25 percentage points in the primary outcome criteria between the study groups at a two-sided significance level of 5% with 80% power, a group sample size of 60 patients is required. Considering an expected dropout rate of 5%, a total of 128 patients will be included in the study. Additionally, a learning sample of 5 patients will be incorporated to allow the surgeons to familiarize themselves with the novel prototype software.

#### Statistical analysis

The statistical analysis will be performed using the statistical software SAS (SAS Institute Inc., Cary, NC, USA) and R (R Foundation for Statistical Computing, Vienna, Austria). The primary efficacy endpoint will be assessed based on the intention-to-treat (ITT) population. Therefore, all patients will be considered as randomized in the analysis. A two-sided Chi-square test at a 0.05 level of significance will be used for the statistical confirmatory analysis. Ninety-five percent confidence intervals will be provided for the difference in proportions and the estimated risk ratio. For the purpose of sensitivity analysis, per protocol- and complete case analysis of the primary endpoint will be conducted and additional missing-value replacement strategies will be employed. Analysis of secondary endpoints will be conducted as appropriate.

## Discussion

Periprosthetic or bony impingement has been correlated to dislocation, increased wear, and reduced postoperative functionality with pain and/or decreased ROM in total hip arthroplasty (THA). Choosing a correct combined orientation of the acetabular cup and femoral stem will yield a maximized, stable range of motion (ROM) and will reduce the risk of dislocation [1,2,5,10,11,25,26]. Following the concept of "femur first", the orthopaedic surgeon has to transfer the orientation of the stem relative to the cup intraoperatively. However, the orthopaedic surgeon faces the intraoperative problem that there is not only a linear correlation between cup anteversion and stem antetorsion, but also dependencies between cup abduction and the neck-to-shaft angle of straight, non-modular stems [11,26]. Moreover, during insertion into the medullary canal, stems of any geometry follow the natural anterior bow of the proximal femur, which creates a deviation between the femoral shaft and the mechanical axis in a sagittal projection; this has been described as "Femoral Tilt" (FT). Re-calculating the compliant component positions according to the concept of combined anteversion with and without the influence of FT revealed that the zone of compliance could differ by more than 200% [27]. Likewise, the so called "Pelvic Tilt" (PT), defined as the angle between this anterior pelvic plane and a vertical line in the standing position, has been reported to have a significant impact on the acetabular cup alignment values, and thereby the final functional anteversion in computer- assisted THA [28,29]. In such a situation, a complex interaction between neck-to-shaft angle, FT, PT, and antetorsion of the stem determine how the cup should be positioned. Surgeons following the concept of "femur first" in THA without the use of intraoperative navigation systems so far estimate femoral antetorsion by visual judgement against the axis of the thigh. However, considerable inaccuracies have been demonstrated when prospectively comparing the operating surgeon's intraoperative estimations of femoral anteversion to postoperative CT scans [7,15]. These findings confirm what has been demonstrated in the past in similar studies: The wide variety of bony anatomical structures and its influence through the adjacent joints regularly leads to an intraoperative false estimation of component position and angularity when visual judgement is used during THA. This happens both to experienced and less-experienced surgeons. Many aspects additionally influencing component position still remain to be taken into account by the surgeon intraoperatively, bearing in mind the individual patient's needs and demands. In such a situation, computer assisted surgery offers a chance to concentrate on this essential goal of the operation [16].

The purpose of the study presented in this article is to compare the clinical and radiographic outcomes of two THA techniques: Minimally invasive, computer-navigated femur first (FF) THA, and a conventional minimally invasive technique. It is our hypothesis that computer-navigated FF THA will lead to an increased ROM without bony or periprosthetic impingement. We also hypothesize that minimally invasive, computer-navigated FF THA leads to better prosthesis positioning and better clinical outcome. The results of this study will be presented as soon as they become available.

## Competing interests

The authors declare that they have no competing interests.

## Authors' contributions

TR and SG originated the idea for the study, led on its design, and will supervise the project. MH, CB, JG were co-applicants on the successful funding proposal. TR, SG, MH, MWo, LD, MWe, NE, CB, PL, RS, MS, ES and JG participated in the design of the study and in developing the novel software protocol and the research protocols. TS and KU provided statistical consultation. TR, SG, MH, CB, MWo, RS, PL, ES and JG will coordinate the trial and are responsible for data acquisition. All authors read and corrected draft versions of the manuscript and approved the final manuscript.

## Pre-publication history

The pre-publication history for this paper can be accessed here:

http://www.biomedcentral.com/1471-2474/12/192/prepub
